# Successful clozapine rechallenge after neutropenia using lithium carbonate : a case report

**DOI:** 10.1192/j.eurpsy.2022.818

**Published:** 2022-09-01

**Authors:** D. Khattech, U. Ouali, N.E.H. Dridi, A. Aissa, A. Ouertani, R. Jomli

**Affiliations:** Razi Hospital, Psychiatry A, Manouba, Tunisia

**Keywords:** schizophrénia, clozapine, Lithium

## Abstract

**Introduction:**

Clozapine is widely known as the drug of choice in treating refractory schizophrenia. However, clozapine prescription requires close clinical and biological monitoring to prevent harmful side effects like agranulocytosis, neutropenia and myocarditis.

**Objectives:**

To show the benefits of lithium carbonate in the clozapine rechallenge of a patient with neutropenia under clozapine.

**Methods:**

We present the clinical case of a patient who developed neutropenia under clozapine, we rechallenged with clozapine after lithium treatment to stimulate hematopoietic functions.

**Results:**

A 42-year-old man diagnosed with refractory schizophrenia, under clozapine for 11 years with a good clinical response at a dosage of 500mg per day (clozapine serum level 328ng/ml), developed a neutropenia (BCC at 840/mm^3^) within an interval of 2 months. Clozapine treatment was suspended and the patient presented a severe psychotic relapse requiring hospitalization. During hospitalization the patient remained symptomatic under haloperidol 15mg daily. At week 3 of clozapine cessation, neutrophil count reached 1510/mm^3^. After week 4 we introduced lithium carbonate and while reaching 500mg per day we observed an increase in the neutrophil count to 4850/mm^3^. We rechallenged with clozapine at week 12 after a poor clinical response, with incremental dosage to 150mg per day in 17 weeks. The blood cell count did not show any abnormalities and the patient had a good clinical response up to 11 months after the clozapine rechallenge.

**Conclusions:**

Despite the lack of guidelines assessing clozapine rechallenge after neutropenia, the use of lithium carbonate may be considered to stimulate hematopoietic functions.

**Disclosure:**

No significant relationships.

